# Functional Visual Loss in a Young Patient With Systemic Lupus Erythematosus

**DOI:** 10.7759/cureus.20513

**Published:** 2021-12-19

**Authors:** Abbas Abd Hamid, Norzila Zakaria, Nurul Ain Masnon, Julieana Muhammed, Wan-Hazabbah Wan Hitam

**Affiliations:** 1 Department of Ophthalmology and Visual Science, School of Medical Sciences, University Sains Malaysia, Kubang Kerian, MYS; 2 Department of Psychiatry, School of Medical Sciences, University Sains Malaysia, Kubang Kerian, MYS

**Keywords:** retrobulbar optic neuritis, systemic lupus erythematosus, visual evoked potential, major depressive disorder, functional visual loss

## Abstract

We describe a rare case of a patient with systemic lupus erythematosus (SLE) with functional visual loss (FVL). A 30-year-old female had blindness in the left eye due to multiple episodes of optic neuritis with underlying SLE. She presented with blurred vision in the right eye after an upper respiratory tract infection. The visual acuity in the right eye was 6/24, while the left eye had no light perception. The right eye optic nerve function tests were within normal limits. There was a positive relative afferent pupillary defect in the left eye. Fundoscopy showed left optic atrophy, while the right fundus was normal. The patient was treated according to a diagnosis of right retrobulbar optic neuritis. However, despite a course of intravenous methylprednisolone, her right visual acuity deteriorated to light perception. A magnetic resonance imaging (MRI) scan of the brain and orbit was normal. The visual evoked potential (VEP) in the right eye was also normal. The patient was suspected of having FVL and was referred to a psychiatrist. She was diagnosed with major depressive disorder after a full psychiatric assessment.

## Introduction

Functional visual loss (FVL) is a decrease in visual acuity or a field defect in the absence of organic pathology. The prevalence of FVL is around 5%-12% in neuro-ophthalmology clinics [1]. It is suspected when patient symptoms are not consistent with the test results.

Functional visual loss can be the earliest presentation of psychiatric disorder in a patient with SLE. Recognizing this as part of psychiatric manifestations is important in order to provide the appropriate treatment and avoid unnecessary further advanced treatment complications. Identification of this problem may help the hidden psychosocial aspect in these patients.

Our patient had underlying systemic lupus erythematosus (SLE) with multiple comorbidities and only one seeing eye. In such a challenging case, it is mandatory to rule out sight-threatening conditions and perform the necessary investigations.

## Case presentation

A 30-year-old female presented with blurred vision in the right eye after an upper respiratory tract infection. She described it as a generalized blurring of vision involving the whole quadrant. She had no eye movement pain, photophobia, color vision changes, or neurological deficit. She had been diagnosed with SLE at the age of eight years when she developed mucocutaneous vasculitis, lupus nephritis, and cerebral lupus. She developed sagittal sinus thrombosis with a positive antiphospholipid antibody. She was treated with warfarin and was followed up regularly by the rheumatology team. Her SLE condition was stable, and her hydroxychloroquine and systemic corticosteroids were discontinued. However, at the age of 15 years, she developed paraplegia and a neurogenic bladder due to transverse myelitis. This was followed by recurrent episodes of optic neuritis in the left eye. Her paraplegia remained, and she used a suprapubic catheter for her neurogenic bladder. She became dependent on her right eye after the left eye became completely blind by the age of 20 years.

The visual acuity in the right eye was 6/24, while the left eye had no light perception. The right optic nerve function tests were within normal limits, and the left eye showed a positive relative afferent pupillary defect. Both anterior segments were unremarkable, with normal intraocular pressure. Fundoscopy showed left optic atrophy, while the right fundus was normal. The patient’s extraocular muscle movement was normal. She had bilateral lower limb hyperreflexia and paraplegia, with a power of 1/5. The other cranial nerves were normal, and there were no signs of cerebellar dysfunction. Other systemic examinations were unremarkable.

**Figure 1:**
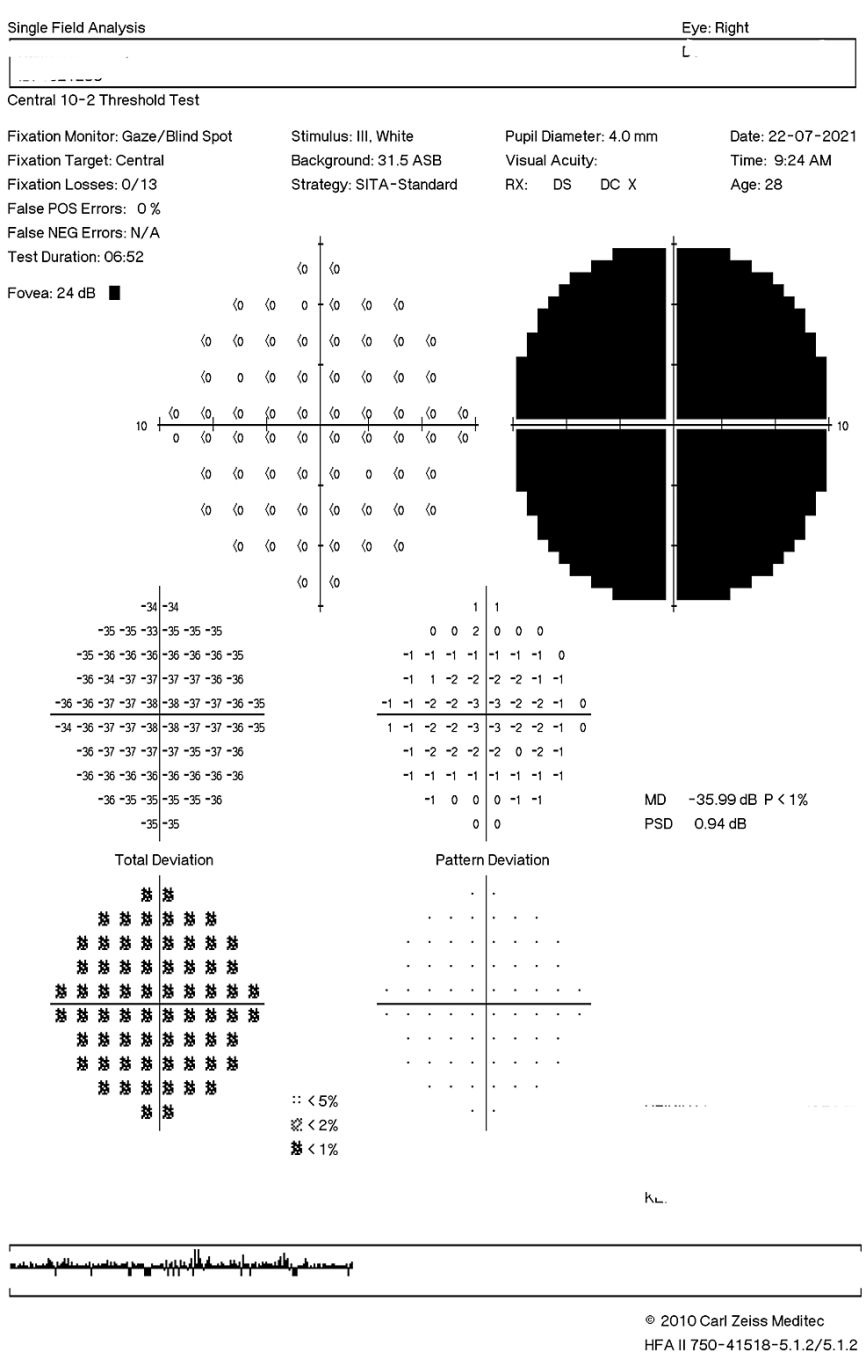
Humphrey Visual Field 10-2 testing of the right eye showed generalized depression

**Figure 2:**
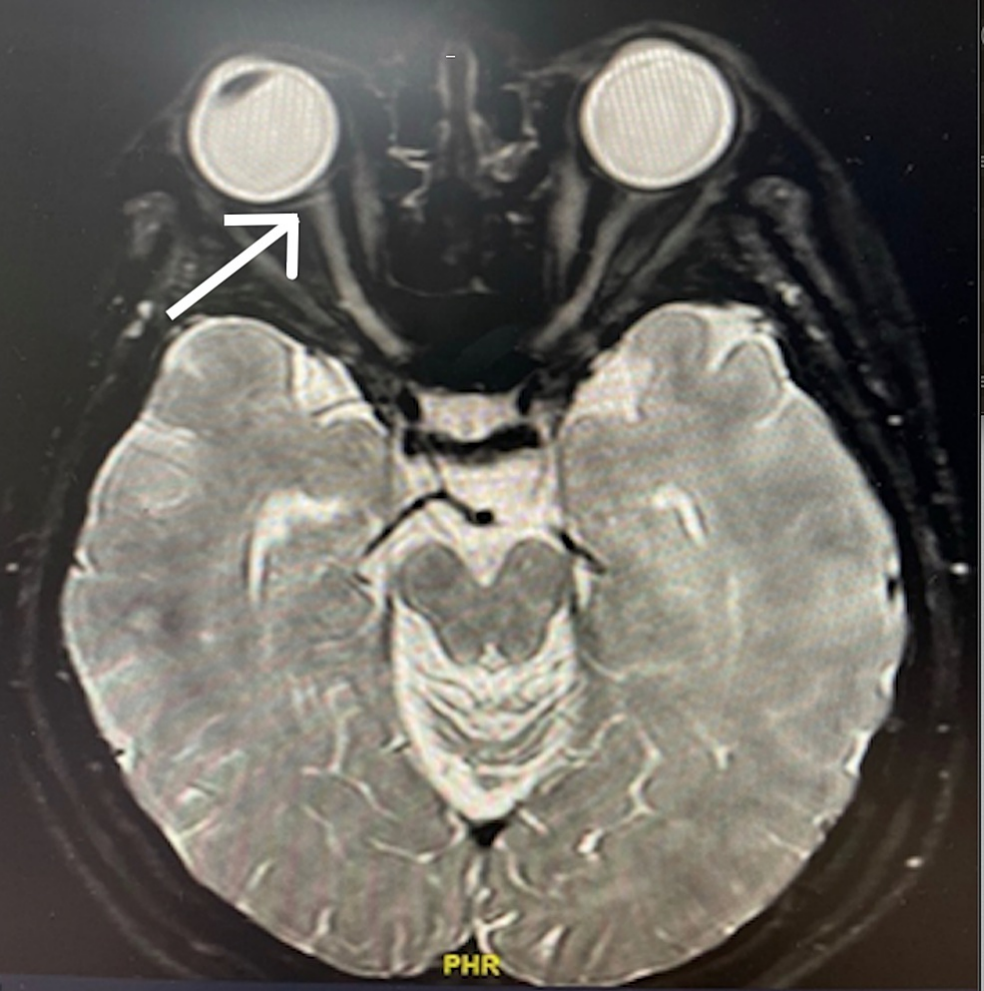
MRI of the brain and orbit White arrow: normal optic nerve, with no enhancement noted

Humphrey Visual Field 10-2 testing of the right eye showed generalized depression (Figure 1). The blood investigations, including full blood count, renal profile, liver function test, erythrocyte sedimentation rate, and C-reactive protein, and complement levels were within normal limits. Infection screening for tuberculosis, syphilis, toxoplasmosis, herpes, and cytomegalovirus was negative. Anti-aquaporin-4 and anti-myelin oligodendrocyte glycoprotein antibodies were also negative. Brain magnetic resonance imaging (MRI) and orbit scans showed normal bilateral optic nerves. MRI of the brain and orbit revealed a normal right optic nerve without any enhancement of the optic nerve sheath (Figure 2).

The initial diagnosis was right retrobulbar optic neuritis, and the patient was started on high-dose intravenous methylprednisolone 250 mg four times a day for a total of three days. However, the visual acuity in the right eye worsened to the perception of hand movement before dropping further to light perception. After referral to the neurology team for further evaluation, the patient was planned for plasma exchange because of a drastic drop in vision despite intravenous methylprednisolone. However, we noticed that she was able to move around without assistance and use her mobile phone for texting. Further assessment of her subjective refraction with a fogging lens of +2.00 DS and a plano lens was performed. Variable results were obtained during the procedure, and they were inconsistent with the patient’s condition. A visual evoked potential (VEP) test showed a normal P100 wave and amplitude for the right eye. The P100 latency of the right eye was also normal (Table 1).

**Table 1:** Visual evoked potential (VEP) test Lat - latency AMP - amplitude LT-RT - left to right

Left eye	Lat N75 (ms)	Lat 2 P100 (ms)	Lat 3 N145 (ms)	PP AMP 75–100 (uV)
M1	1:N75	1:P100	1:N145	1:N75 P100
	102	134	173	2.55
M2	1:P100	2:P100	2:N145	2:N75 P100
	134	134	174	2.82
M3	3:N75	3:P100	3:N145	3:N75 P100
	103	135	174	1.72
Right eye	Lat N75 (ms)	Lat 2 P100 (ms)	Lat 3 N145 (ms)	PP AMP 75–100 (uV)
M1	4:N75	4:P100	4:N145	4:N75 P100
	92	114	140	3.69
M2	5:N75	5:P100	5:N145	5:N75 P100
	93.5	116	140	4.33
M3	6:N75	6:P100	6:N145	6:N75 P100
	92	115	139	3.75
Interocular measurements		LatDiff LT-RT (ms)		PP AMP Ratio LT-RT (%)
MO		1:P100 4:P100		1:N75 P100 4:N75 P100
		-20		145

A diagnosis of FVL was entertained. The patient was referred to the psychiatry team for further assessment. The psychiatric assessment confirmed that the patient was experiencing a depressed mood, feelings of worthlessness, poor concentration, sleep disturbances, and psychomotor retardation. She fulfilled the criteria for a diagnosis of major depressive disorder. She was started on oral escitalopram 10 mg and clonazepam 0.5 mg. On follow-up at three months, her best visual acuity was 6/24. She was advised to attend for regular follow-up.

## Discussion

Most reported FVL cases in the literature are post-traumatic cases. To the best of our knowledge, no case of FVL in chronic medical illnesses has been reported, especially in patients with SLE. SLE caused a significant burden and morbidity to our patient, and in this case, it was a presenting sign of a psychiatric illness.

The most common mood disorder in patients with lupus is depression, ranging from 11% to 39% [2]. The most frequently reported depressive symptoms in lupus are fatigue and weakness (88%-90%), irritability (82.3%), somatic complaints (76%), sleep disturbances (70%), and sadness (29%-73%) [3]. In our patient’s case, FVL was the presenting symptom. The pathogenesis and role of autoantibodies are not well understood. However, a systematic review showed that psychiatric symptoms lead to medical nonadherence and thus to disease-related complications [4].

Reduced visual acuity and visual fields are the most common presentations of FVL. A reported case series of FVL in idiopathic intracranial hypertension included 17 cases, of which 80% had psychosocial and psychiatric comorbidities [1].

FVL is never a diagnosis of exclusion. A positive finding must be made before making a diagnosis. A high index of suspicion is the key to diagnosis. The visual acuity test should include reading the eye chart from the bottom upward. A fogging test, in which the good eye is fogged so that the patient’s remaining vision will reflect the function of the bad eye, can also be performed. It was difficult in our case since the patient had only one seeing eye. An automated visual field can map out a concentric loss of peripheral vision, a cloverleaf pattern, or hemianopsia [5]. VEP is the only objective measurement and is very helpful in detecting FVL. VEP is also able to differentiate other differential diagnoses [6].

## Conclusions

In conclusion, FVL can be the earliest presentation of a psychiatric disorder in a patient with SLE. Recognizing this as part of a psychiatric manifestation is important to provide the appropriate treatment and avoid unnecessary treatment complications. Identification of this problem may help uncover hidden psychosocial aspects in these patients.

## References

[1] Ney JJ, Volpe NJ, Liu GT, Balcer LJ, Moster ML, Galetta SL (2009). Functional visual loss in idiopathic intracranial hypertension. Ophthalmology.

[2] Meszaros ZS, Perl A, Faraone SV (2012). Psychiatric symptoms in systemic lupus erythematosus: a systematic review. J Clin Psychiatry.

[3] Palagini L, Mosca M, Tani C, Gemignani A, Mauri M, Bombardieri S (2013). Depression and systemic lupus erythematosus: a systematic review. Lupus.

[4] Azizoddin DR, Zamora-Racaza G, Ormseth SR (2017). Psychological factors that link socioeconomic status to depression/anxiety in patients with systemic lupus erythematosus. J Clin Psychol Med Settings.

[5] Smith TJ, Baker RS (1987). Perimetric findings in functional disorders using automated techniques. Ophthalmology.

[6] Lim SA, Siatkowski RM, Farris BK (2005). Functional visual loss in adults and children patient characteristics, management, and outcomes. Ophthalmology.

